# Effects of Ni Doping on Thermoelectric Properties of Chalcopyrite

**DOI:** 10.3390/ma18122738

**Published:** 2025-06-11

**Authors:** Hyeokmin Kwon, Il-Ho Kim

**Affiliations:** Department of Materials Science and Engineering, College of Engineering, Korea National University of Transportation, Chungju 27469, Republic of Korea; hmkwon2525@naver.com

**Keywords:** thermoelectric, chalcopyrite, doping

## Abstract

Chalcopyrite (CuFeS_2_) has attracted interest as a thermoelectric material due to its narrow bandgap and its ability to tailor its carrier concentration through doping. In this study, we investigated the effects of Ni^2+^ substitution at Cu^+^ sites in chalcopyrite (Cu_1−x_Ni_x_FeS_2_) on its structural, microstructural, and thermoelectric properties. Samples were synthesized using mechanical alloying followed by hot pressing to ensure high-density compaction. X-ray diffraction analysis confirmed the formation of the tetragonal chalcopyrite phase without detectable secondary phases. The observed reduction in lattice parameters with increasing Ni content provided evidence of successful Ni incorporation at Cu sites within the chalcopyrite structure. Microstructural analysis and elemental mapping further supported the uniform distribution of Ni within the chalcopyrite matrix. Thermoelectric property measurements revealed that Ni-doped chalcopyrite exhibited n-type conduction. As the Ni concentration increased, the carrier concentration and electrical conductivity increased significantly, with Cu_0.92_Ni_0.08_FeS_2_ achieving the highest electrical conductivity of 2.5 × 10^4^ Sm^−1^ at 723 K. However, the absolute value of the Seebeck coefficient decreased with increasing Ni doping, following the expected trade-off between electrical conductivity and thermopower. The optimized composition, Cu_0.96_Ni_0.04_FeS_2_, exhibited the highest thermoelectric performance, with a power factor of 0.50 mWm^−1^K^−2^ and a maximum dimensionless figure of merit (ZT) of 0.18 at 623 K. Compared to undoped chalcopyrite, these enhancements represent a 43% increase in power factor and a 50% improvement in ZT.

## 1. Introduction

Thermoelectric power generation, which directly converts waste heat into electrical energy, has attracted attention as an energy-efficient and environmentally friendly technology. Thermoelectric materials such as Bi_2_Te_3_-based alloys [1,2,3] and PbTe-based alloys [4,5,6] have been widely used; however, the scarcity of Te and the toxicity of Pb highlight the need for economical and eco-friendly thermoelectric materials. Chalcopyrite (CuFeS_2_) stands out as a material composed of abundant and low-toxicity elements. Chalcopyrite adopts a tetragonal structure with the space group $I\bar{4}2d$ [7,8,9,10]. CuFeS_2_ is an n-type semiconductor with intrinsic sulfur vacancies and a bandgap of 0.53 eV [11,12,13]. Considering the charge balance of chalcopyrite, it may exist as Cu^+^Fe^3+^S_2_^2−^, Cu^2+^Fe^2+^S_2_^2−^, or in a mixed state [14,15]. However, due to the energetic instability of Cu^2+^ coordinated with chalcogen ligands, chalcopyrite predominantly adopts the Cu^+^Fe^3+^S_2_^2−^ state [16].

Electron spins can influence the transport properties of the antiferromagnetic CuFeS_2_, and it is known that the tetragonal structure of chalcopyrite undergoes a phase transition at a Néel temperature of 823 K [9], leading to disorder between Cu and Fe ions [17]. Studies have reported the partial substitution (doping) of Cu sites in chalcopyrite with metal cations such as In^+/3+^ [16], Ag^2+^ [18], Cd^2+^ [19], Zn^2+^ [20], Co^2+^ [21], and Mn^2+^ [22] to control thermoelectric properties. Ge et al. [16] demonstrated that doping with In^+^ cations, which replace Cu^+^ in the sublattice with 5s^2^ lone-pair electrons, significantly reduced lattice thermal conductivity, thereby improving thermoelectric performance. Ge et al. [18] achieved enhanced thermoelectric properties by tuning (reducing) the bandgap through Ag doping, thereby increasing carrier mobility. Similarly, Ge et al. [19] reported that substituting Cd for Cu created ${Cd}_{Cu}^{\cdot }$ point defects and ${Fe}_{Cu}^{\cdot \cdot }$ antisite defects, contributing to improved electrical conductivity and enhanced thermoelectric performance. Xie et al. [20] proposed that Zn doping effectively increases carrier concentration, acting as a donor to improve thermoelectric properties. Berthebaud et al. [21] found that Co substitution at Cu sites significantly increases electrical conductivity and the power factor. Lefevre et al. [22] claimed that Mn exhibited doping effects similar to Co, with the local disorder induced by Mn reducing thermal conductivity.

In this study, Ni^2+^ was doped into Cu^+^ sites in chalcopyrite to induce excess electron supply, and changes in its charge transport properties were investigated. The electrical and thermal parameters were evaluated, and the results were compared with those of chalcopyrite doped with other elements reported in the literature.

## 2. Experimental Procedure

Cu (purity: 99.9%, particle size: <45 µm), Ni (purity: 99.9%, particle size: <73 µm), Fe (purity: 99.9%, particle size: <53 µm), and S (purity: 99.99%, particle size: <75 µm) powders were used to prepare Cu_1−x_Ni_x_FeS_2_ (x = 0.04, 0.06, and 0.08) compositions. The precursor powders were mixed and placed in stainless steel jars with stainless steel balls for mechanical alloying (MA; Pulverisette5, Fritsch, Pittsboro, NC, USA). The process was conducted under an Ar atmosphere at a rotational speed of 350 rpm for 12 h. The alloyed powders were consolidated into Ni-doped chalcopyrite compounds by hot pressing (HP; JM-HP20, Jungmin, Seoul, Republic of Korea) under vacuum at 70 MPa and 773 K for 2 h. The MA and HP processing parameters were optimized based on a previous study [23] to ensure proper synthesis and sintering.

Phase analysis and lattice constant measurements of the MA-synthesized powders and HP-consolidated samples were performed using X-ray diffraction (XRD; D8-Advance, Bruker, Billerica, MA, USA) with Cu Kα radiation, followed by Rietveld refinement (TOPAS). The fractured surfaces of the HP samples were examined using scanning electron microscopy (SEM; Prisma E, Thermo Fisher Scientific, Waltham, MA, USA), and the elemental distributions were analyzed using energy-dispersive spectroscopy (EDS; Quantax200, Bruker, Billerica, MA, USA). The sintered specimens were sectioned into rectangular parallelepipeds with dimensions of 3 × 3 × 9 mm^3^ for Seebeck coefficient and electrical conductivity measurements, and into discs with a diameter of 10 mm and a thickness of 1 mm for thermal conductivity and Hall effect measurements. Carrier concentration and mobility were measured at room temperature using the van der Pauw method with a data acquisition system (TC2110, Keithley Instruments, Solon, OH, USA) equipped with a Hall interface (7065, Keithley Instruments, Solon, OH, USA) and an electromagnet (HV4H, Walker Scientific, Worcester, MA, USA). The Seebeck coefficient (α) and electrical conductivity (σ) were measured under a He atmosphere using the DC four-probe method with a ZEM-3 instrument (Advance Riko, Yokohama, Japan). Thermal diffusivity (D) was measured under vacuum using the laser flash method with the TC-9000H equipment (Advance Riko, Yokohama, Japan). Thermal conductivity (κ = D · cₚ · d) was calculated from specific heat capacity (cₚ) and density (d). Finally, the power factor (PF = α^2^ · σ) and dimensionless figure of merit (ZT = PF · κ^−1^ · T) were evaluated over a temperature (T) range of 323–723 K.

## 3. Results and Discussion

**Figure 1:**
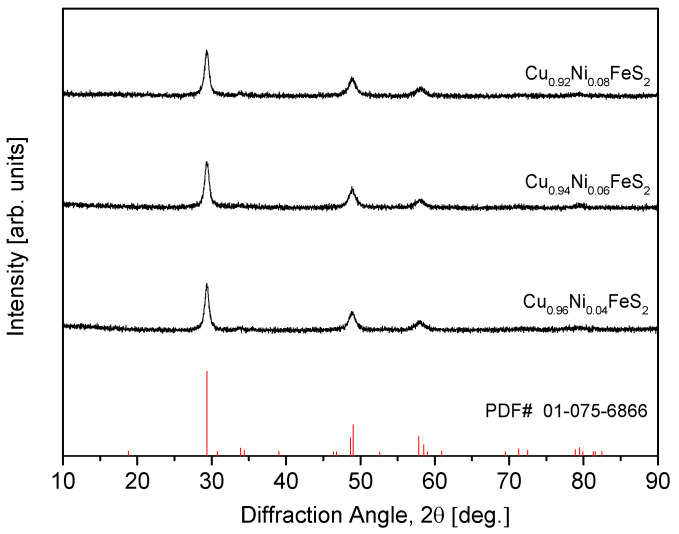
XRD patterns of Cu_1−x_Ni_x_FeS_2_ powders synthesized using mechanical alloying.

Figure 1 presents the XRD patterns of Cu_1−x_Ni_x_FeS_2_ powders synthesized via MA. All samples exhibited a single-phase tetragonal structure consistent with the standard chalcopyrite phase (PDF# 01-075-6866), indicating successful formation without the presence of detectable secondary phases. Despite the partial substitution of Cu^+^ by Ni^2+^, the chalcopyrite structure was preserved, suggesting that Ni was successfully incorporated into the lattice without exceeding the solubility limit within the examined doping range. The observed broadening of diffraction peaks across all samples can be attributed to the severe plastic deformation, grain refinement, and internal strain induced by the high-energy impacts during MA. This behavior is typical for mechanically alloyed materials. Notably, the solubility limit for Ni in the Cu_1−x_Ni_x_FeS_2_ system was not reached up to x = 0.08. This is particularly significant when compared with previously reported solubility limits for other dopants in chalcopyrite structures. For instance, Ge et al. [16,18,19] reported solubility limits of x = 0.08 for Ag, x = 0.02 for Cd, and x = 0.08 for In in Cu_1−x_Ag_x_FeS_2_, Cu_1−x_Cd_x_FeS_2_, and Cu_1−x_In_x_FeS_2_, respectively, synthesized via encapsulated melting. In each case, exceeding the solubility limit resulted in the appearance of secondary phases such as Ag_2_S, FeS_2_/CdS, and CuInS_2_. Similarly, Xie et al. [20] observed a solubility limit of x = 0.03 for Zn in Cu_1−x_Zn_x_FeS_2_ synthesized by vacuum melting, with ZnS forming at higher doping levels. Berthebaud et al. [21] also reported a solubility limit of x = 0.08 for Co in Cu_1−x_Co_x_FeS_2_ prepared via cold pressing and solid-state reaction, but multiple secondary phases including CoS_2_ emerged at x = 0.1. In comparison, the Ni-doped CuFeS_2_ samples in the present study maintained a single-phase chalcopyrite structure up to x = 0.08 without any secondary phases, even under the high-energy conditions of MA. This suggests that Ni has a relatively broad solubility range within the CuFeS_2_ lattice and that MA is an effective synthesis technique for promoting dopant incorporation while suppressing secondary phase formation. Furthermore, the absence of secondary phases even at the highest doping level confirms the thermodynamic compatibility and structural stability of Ni within the chalcopyrite matrix.

**Figure 2:**
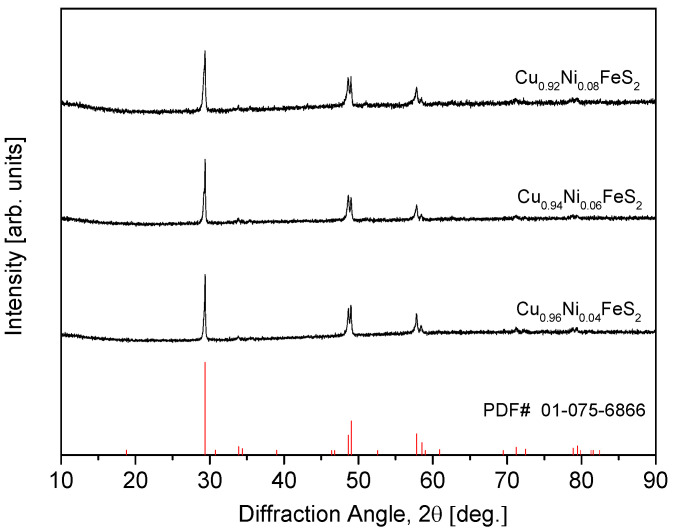
XRD patterns of Cu_1−x_Ni_x_FeS_2_ specimens sintered using hot pressing.

The XRD analysis was conducted on bulk Cu_1−x_Ni_x_FeS_2_ samples consolidated via the HP of the MA powders, and the results are presented in Figure 2. The diffraction patterns of all sintered specimens were consistent with the tetragonal chalcopyrite structure, with no evidence of secondary phases, indicating that the structural integrity of the chalcopyrite phase was preserved throughout the HP process. This outcome demonstrates that the applied HP conditions were thermodynamically and kinetically favorable for stabilizing the single-phase chalcopyrite structure, even in the presence of Ni substitution, which can potentially lead to phase separation in other doped systems. Moreover, the XRD patterns revealed the noticeable sharpening of diffraction peaks compared to the as-milled powders. This peak sharpening is indicative of increased crystallinity and grain coarsening due to the high-temperature sintering, which facilitates atomic diffusion and grain boundary migration. Additionally, the reduction in peak broadening suggests the partial relaxation of microstrain and a decrease in crystal defects introduced during MA.

Table 1 summarizes the relative densities and lattice parameters of the Cu_1−x_Ni_x_FeS_2_ samples synthesized by mechanical alloying followed by hot pressing (MA–HP). The relative density was calculated based on the theoretical density of chalcopyrite (4.3 gcm^−3^) [24], with all samples exhibiting high relative densities in the range of 98.3–99.3%. These values indicate that the HP process was highly effective in achieving dense compaction, with minimal porosity across all compositions. The lattice parameters of undoped CuFeS_2_ were measured as a = 0.5292 nm and c = 1.0438 nm, yielding a tetragonal axial ratio c/a = 1.9724 [23]. Upon the substitution of Cu^+^ by Ni^2+^, a systematic contraction of the lattice was observed: the a-axis decreased from 0.5291 to 0.5287 nm, the c-axis from 1.0435 to 1.0418 nm, and the c/a ratio slightly declined from 1.9722 to 1.9705. This reduction in both lattice parameters and tetragonality can be attributed to the smaller ionic radius of Ni^2+^ (69 pm) [25] relative to that of Cu^+^ (74 pm) [26], confirming the successful incorporation of Ni into the Cu sites of the chalcopyrite structure. These results are in agreement with previous studies. For instance, Ge et al. [16,18] observed an increase in lattice parameters when larger ions such as In^+^ and Ag^+^ were substituted at Cu sites in Cu_1−x_In_x_FeS_2_ and Cu_1−x_Ag_x_FeS_2_, due to their larger ionic radii compared to Cu^+^. In contrast, Xie et al. [20] reported that, in Cu_1−x_Zn_x_FeS_2_, lattice constants increased only up to the solubility limit of Zn and then plateaued due to the formation of antisite defects involving Fe and Cu, in line with the atomic radius trend (Fe < Cu < Zn). Furthermore, Aliyev et al. [27] found that the substitution of Fe^3+^ by Ni^2+^ in chalcopyrite did not result in significant changes in lattice constants, highlighting that the site of substitution critically affects the structural response. Therefore, the observed contraction in lattice parameters in the present study clearly supports the substitution of Cu^+^ by Ni^2+^, and not Fe^3+^, within the chalcopyrite lattice.

**Figure 3:**
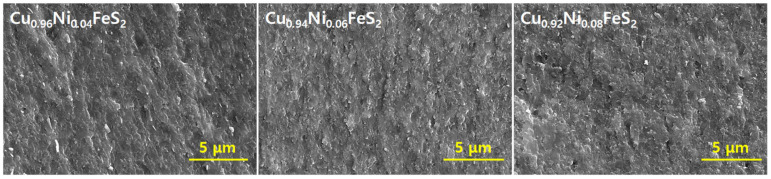
SEM images of fractured surfaces for Cu_1−x_Ni_x_FeS_2_.

Figure 3 presents the fractured surface morphologies of the hot-pressed Cu_1−x_Ni_x_FeS_2_ samples, as observed via SEM. The images reveal well-consolidated microstructures across all compositions, consistent with the high relative densities shown in Table 1, confirming the effectiveness of the HP process in achieving dense bulk materials. Notably, no apparent microstructural anomalies or secondary phases were observed with increasing Ni content up to x = 0.08, indicating that Ni incorporation did not compromise the phase stability of the chalcopyrite matrix. This aligns with the XRD results, which showed no secondary phase formation, thereby confirming the high solubility of Ni within the Cu sublattice under the applied synthesis conditions. Crystallite sizes, derived from Rietveld refinement analysis, showed a significant decrease from 85 nm at x = 0.04 to 36 nm at x = 0.08. This trend suggests that Ni doping introduces localized lattice strain or disorder within the CuFeS_2_ structure, which can suppress grain growth during sintering. The ionic radius mismatch between Ni^2+^ and Cu^+^ likely contributes to lattice distortion, thereby hindering crystallite coarsening and promoting grain refinement.

**Figure 4:**
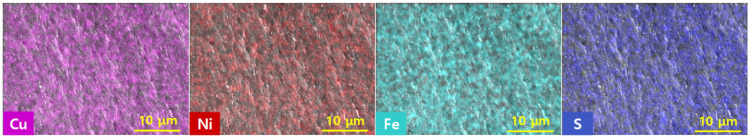
EDS elemental maps of Cu_0.96_Ni_0.04_FeS_2_.

Figure 4 shows the two-dimensional EDS elemental maps for the Cu_0.96_Ni_0.04_FeS_2_ sample, providing insight into the spatial distribution of Cu, Ni, Fe, and S within the chalcopyrite matrix. The EDS maps confirm a homogeneous distribution of all constituent elements, indicating that Ni was uniformly incorporated into the lattice without any observable segregation or clustering. This compositional uniformity stands in contrast to the common issues associated with conventional high-temperature melting techniques, where elements with high vapor pressures—particularly sulfur—can volatilize, and dopants such as Ni may segregate due to insufficient diffusion or rapid cooling rates. These challenges often lead to inhomogeneous elemental distributions, secondary phase formation, and compromised thermoelectric performance. The use of mechanical alloying followed by hot pressing in this study effectively mitigated these issues. Mechanical alloying ensures the thorough mixing and alloying of the starting powders at room temperature, thereby minimizing volatilization losses. Subsequent high-temperature hot pressing promotes densification while maintaining compositional uniformity. The uniform elemental distribution achieved via this solid-state synthesis route highlights the advantage of MA–HP for producing phase-pure, compositionally homogeneous thermoelectric materials.

**Figure 5:**
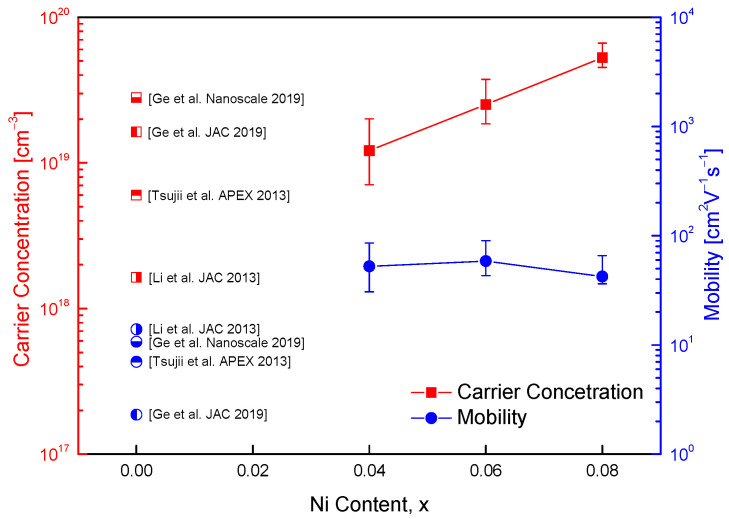
Charge transport properties of Cu_1−x_Ni_x_FeS_2_. For comparison, the carrier concentration and mobility values of undoped chalcopyrite compounds prepared by various processing methods were presented [9,10,18,19].

Figure 5 illustrates the carrier concentration and Hall mobility of Cu_1−x_Ni_x_FeS_2_ samples, indicating the influence of Ni substitution on charge transport properties. For context, data from undoped CuFeS_2_ reported in previous studies [9,10,18,19] are also included for comparison. CuFeS_2_ is known to exhibit intrinsic n-type semiconducting behavior due to native defects such as sulfur vacancies and antisite defects. Substitution of divalent Ni^2+^ for monovalent Cu^+^ introduces additional electrons, thereby increasing the electron carrier concentration. In this study, the carrier concentration increased from 7.8 × 10^18^ cm^−3^ for Cu_0.96_Ni_0.04_FeS_2_ to 1.4 × 10^19^ cm^−3^ for Cu_0.92_Ni_0.08_FeS_2_. These values are consistent with previously reported carrier concentrations for undoped CuFeS_2_, which typically range between 1.6 × 10^18^ and 1.6 × 10^19^ cm^−3^ [9,10,18,19], confirming the effectiveness of Ni doping in enhancing electron concentration. In comparison, Ge et al. [18] reported a maximum carrier concentration of 3.7 × 10^18^ cm^−3^ for Ag-doped CuFeS_2_, where Ag^+^ substitutes for Cu^+^. This relatively modest increase is due to the isovalent nature of Ag and Cu, which limits the generation of additional charge carriers. On the other hand, Cd^2+^ doping at the Cu site, as reported by Ge et al. [19], led to a much higher carrier concentration of up to 2.1 × 10^20^ cm^−3^, owing to the aliovalent substitution introducing a significant number of excess electrons. However, such high doping levels can also lead to increased defect scattering and reduced mobility. In the current study, a slight decrease in Hall mobility was observed with increasing Ni content: from 33.5 cm^2^V^−1^s^−1^ for Cu_0.96_Ni_0.04_FeS_2_ to 23.5 cm^2^V^−1^s^−1^ for Cu_0.92_Ni_0.08_FeS_2_. This reduction is attributed to increased impurity scattering introduced by Ni dopants, which disrupt the periodic potential and hinder carrier transport. Nonetheless, these mobility values remain significantly higher than those reported in the literature for both undoped and doped CuFeS_2_ systems, which generally range from 2.3 to 13.9 cm^2^V^−1^s^−1^ [9,10,18,19]. The relatively high mobility observed in this study may be due to improved microstructural homogeneity, as evidenced by the dense microstructure and uniform element distribution. Furthermore, the moderate doping levels and well-controlled MA–HP synthesis likely minimized the formation of defect clusters or secondary phases that typically degrade mobility.

**Figure 6:**
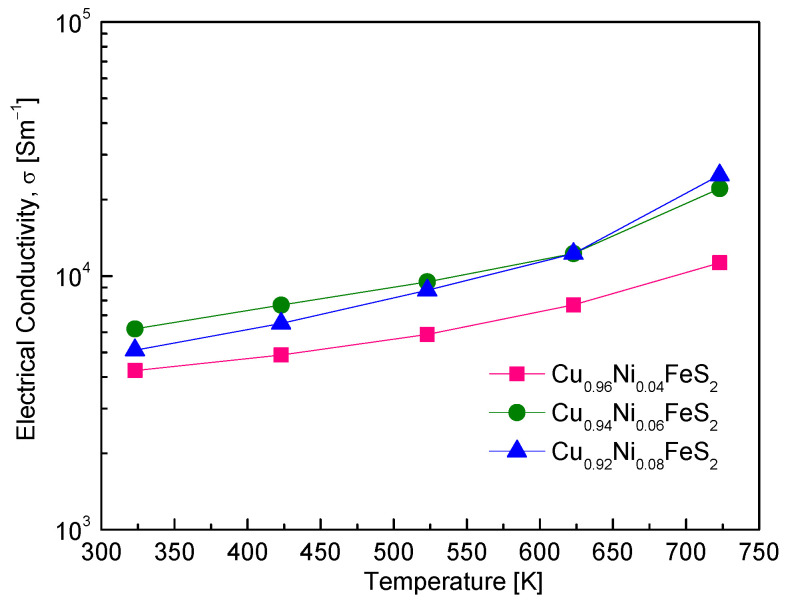
Temperature dependence of electrical conductivity for Cu_1−x_Ni_x_FeS_2_.

Figure 6 illustrates the temperature dependence of electrical conductivity for Cu_1−x_Ni_x_FeS_2_ (x = 0.04–0.08). All samples exhibited semiconducting behavior, with electrical conductivity increasing with rising temperature. This behavior indicates thermally activated charge transport, where thermal excitation promotes electrons across the bandgap into the conduction band, thereby enhancing the electrical conductivity. Ni doping led to an overall increase in electrical conductivity, attributed to the substitution of Ni^2+^ for Cu^+^. This aliovalent substitution introduces additional electrons, increasing the carrier concentration. However, no significant difference in electrical conductivity was observed between x = 0.06 and x = 0.08. This plateau likely results from the competing effects of increased carrier concentration and enhanced carrier scattering at higher doping levels, which reduce carrier mobility. Among the compositions, Cu_0.92_Ni_0.08_FeS_2_ exhibited the highest electrical conductivity, reaching 2.5 × 10^4^ Sm^−1^ at 723 K. Compared to prior studies, the electrical conductivity achieved in this work is notable. Ge et al. [16] reported an increase in electrical conductivity in Cu_1−x_In_x_FeS_2_ from 0.3 × 10^4^ Sm^−1^ (x = 0) to 1.9 × 10^4^ Sm^−1^ (x = 0.08) at 723 K. Similarly, Ge et al. [18,19] found that Ag and Cd doping also enhanced conductivity, with values of 2.0 × 10^4^ Sm^−1^ at 323 K for Cu_0.88_Ag_0.12_FeS_2_ and 1.3 × 10^4^ Sm^−1^ at 300 K for Cu_0.92_Cd_0.08_FeS_2_. However, these doped systems exhibited degenerate semiconductor behavior, characterized by decreasing electrical conductivity with increasing temperature—a behavior associated with metal-like conduction due to a high carrier concentration. A similar degenerate trend was observed by Xie et al. [20] in Zn-doped Cu_1−x_Zn_x_FeS_2_, where the electrical conductivity increased with Zn doping up to 3.0 × 10^4^ Sm^−1^ at 300 K, but decreased to 1.5 × 10^4^ Sm^−1^ at 630 K. Berthebaud et al. [21] also reported behavior associated with metallic conduction in Co-doped Cu_1−x_Co_x_FeS_2_, where the electrical resistivity decreased with temperature. In contrast, the present study’s Ni-doped samples clearly exhibit semiconducting behavior, even at high carrier concentrations. This discrepancy is intriguing and suggests that the electronic structure of Ni-doped chalcopyrite differs from that of other metal-doped chalcopyrites. Based on the carrier concentration range shown in Figure 5, degenerate behavior would typically be expected. However, the persistence of semiconducting behavior, marked by an increase in conductivity with temperature, suggests that intrinsic excitations significantly influence charge transport at elevated temperatures. Ni doping likely shifts the Fermi level closer to the conduction band minimum, enhancing electron population. At higher temperatures, intrinsic excitation across the bandgap could become significant, resulting in a rapid increase in electron concentration and electrical conductivity. This hypothesis is supported by the sharp rise in conductivity observed at elevated temperatures, particularly for Cu_0.92_Ni_0.08_FeS_2_. In thermoelectric materials, the onset of intrinsic excitation is typically accompanied by a decrease in the absolute value of the Seebeck coefficient. Thus, further insights into this behavior can be gained by correlating electrical conductivity with the temperature dependence of the Seebeck coefficient, as discussed in the subsequent sections.

**Figure 7:**
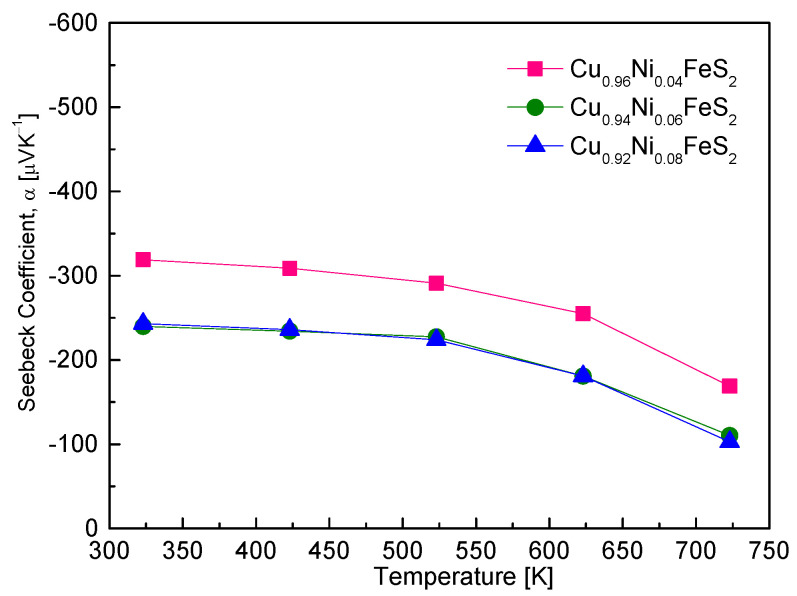
Temperature dependence of Seebeck coefficient for Cu_1−x_Ni_x_FeS_2_.

Figure 7 presents the temperature-dependent Seebeck coefficient of Cu_1−x_Ni_x_FeS_2_ samples. All samples exhibited negative Seebeck coefficients across the entire measured temperature range (323–723 K), confirming their n-type semiconducting behavior. The origin of this behavior lies in the substitution of Ni^2+^ for Cu^+^, which introduces excess electrons and increases the electron carrier concentration, as expected from the following relation [20]: α = (8/3)π^2^k_B_^2^e^−1^h^−2^m*T(π/3n)^2/3^, where k_B_ denotes the Boltzmann constant, e denotes the electron charge, h denotes the Planck constant, m^⁎^ denotes the effective mass of the carriers, and n denotes the carrier concentration [20]. According to this relationship, the Seebeck coefficient is inversely related to the carrier concentration and directly proportional to the temperature and effective mass. In this study, Cu_0.96_Ni_0.04_FeS_2_ demonstrated the largest absolute values of the Seebeck coefficient, ranging from −319 to −169 μVK^−1^, depending on temperature. As Ni content increased, the absolute Seebeck coefficient systematically decreased due to the enhanced carrier concentration. This is consistent with the inverse relationship between the Seebeck coefficient and carrier concentration predicted by the above equation. Furthermore, all Ni-doped samples exhibited a decline in the absolute value of the Seebeck coefficient with increasing temperature, particularly above ~573 K. This trend suggests the occurrence of intrinsic excitation, where thermal energy promotes a significant number of electrons from the valence band to the conduction band, thereby increasing the total carrier concentration and reducing the Seebeck coefficient. The presence of intrinsic transitions also explains the positive temperature dependence of electrical conductivity observed in Figure 6. While degenerate semiconductors often exhibit metallic behavior (decreasing conductivity with increasing temperature), the onset of intrinsic conduction can reverse this trend. The correlation between the rapid decline in Seebeck coefficient and the increase in electrical conductivity at high temperatures strongly supports this interpretation. Ge et al. [18] found that Cu_1−x_Ag_x_FeS_2_ showed a decrease in the absolute value of α with increasing Ag content, attributed to bandgap narrowing and a resulting reduction in the effective mass of electrons. Similarly, Xie et al. [20] reported that, in Cu_1−x_Zn_x_FeS_2_, the Seebeck coefficient decreased with increasing Zn content, reaching −70 μVK^−1^ at 630 K for Cu_0.9_Zn_0.1_FeS_2_, suggesting a complex interplay between enhanced carrier concentration and changes in effective mass due to Zn doping. Berthebaud et al. [21] and Lefevre et al. [22] also observed decreasing Seebeck coefficients with increasing doping levels of Co and Mn, respectively, when doped at the Cu site. These results confirm that metal cation substitution at the Cu site induces n-type conductivity in chalcopyrite and increases the carrier concentration, leading to a reduced absolute value of the Seebeck coefficient. The magnitude of Seebeck coefficient is therefore governed not only by carrier concentration but also by factors like the effective mass and the onset of intrinsic transitions at elevated temperatures.

**Figure 8:**
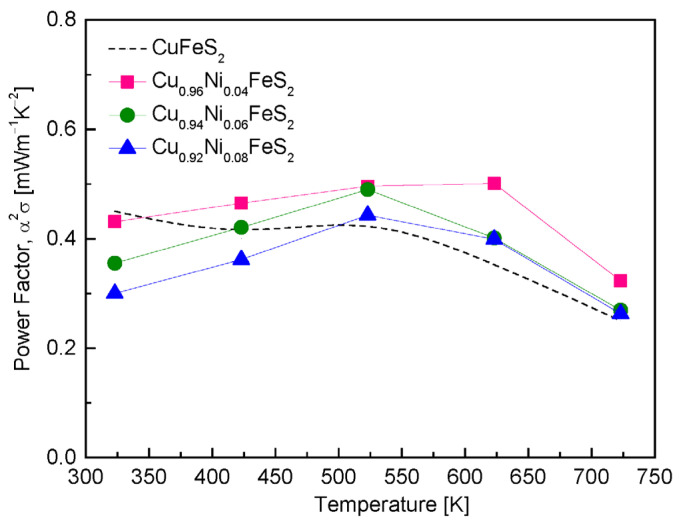
Temperature dependence of power factor for Cu_1−x_Ni_x_FeS_2_. For comparison, the power factor values of undoped chalcopyrite were presented [18].

Figure 8 presents the power factor (PF) of Cu_1−x_Ni_x_FeS_2_, which serves as a critical indicator of thermoelectric performance by combining electrical conductivity and the Seebeck coefficient. In the Ni-doped samples, the PF exhibited a peak within the temperature range of 523–623 K, with Cu_0.96_Ni_0.04_FeS_2_ achieving the highest value of 0.50 mWm^−1^K^−2^ at 623 K. Interestingly, PF values decreased with increasing Ni content, indicating that while Ni doping initially enhances carrier concentration and thereby electrical conductivity, excessive Ni content may lead to detrimental effects such as enhanced charge carrier scattering or a sharp reduction in the Seebeck coefficient. When compared to undoped CuFeS_2_ [18], which showed lower PF values of 0.45 mWm^−1^K^−2^ at 323 K and 0.25 mWm^−1^K^−2^ at 723 K, the Ni-doped composition demonstrated superior performance in the high-temperature region. This suggests that Ni doping effectively boosts thermoelectric efficiency at elevated temperatures, where the conductivity gain compensates for the Seebeck loss. A comparative analysis with other dopants provides further insight into the relative efficacy of Ni doping. Ge et al. [16,18,19] reported significantly higher PF values for other metal substitutions in CuFeS_2_, including 0.74 mWm^−1^K^−2^ for Cu_0.96_In_0.04_FeS_2_, 0.95 mWm^−1^K^−2^ for Cu_0.88_Ag_0.12_FeS_2_, and 0.43 mWm^−1^K^−2^ for Cu_0.92_Cd_0.08_FeS_2_. Among these, Ag doping yielded the highest PF, most likely due to its dual role in enhancing carrier mobility and inducing favorable modifications in the electronic band structure. In contrast, while Cd doping yielded PF values slightly lower than those for Ni at high temperature, it still showed a comparable enhancement over the undoped material. Moreover, Xie et al. [20] reported a peak PF of 0.97 mWm^−1^K^−2^ at 300 K for Cu_0.96_Zn_0.04_FeS_2_—almost double that of the Ni-doped sample at its highest point. This remarkable performance was attributed to a combination of increased electrical conductivity and an enhanced effective mass of charge carriers due to Zn doping. However, as the temperature increased to 630 K, the PF declined to 0.81 mWm^−1^K^−2^, still outperforming the Ni-doped composition. This indicates that Zn doping not only improves PF at room temperature but retains relatively high performance across a broader temperature range. Additionally, Berthebaud et al. [21] found that Cu_0.96_Co_0.04_FeS_2_ exhibited a PF of 0.62 mWm^−1^K^−2^ at 370 K, further underscoring that alternative dopants like Co can offer better low-to-mid temperature PF performance than Ni. The superior PF observed with In, Ag, and Zn doping in other studies suggests that Ni doping, while moderately effective, does not optimize the electronic band structure or carrier scattering mechanisms as effectively as these elements. Overall, although Ni doping improves the PF of CuFeS_2_, especially in the higher-temperature region, it is less effective compared to other metal dopants.

**Figure 9:**
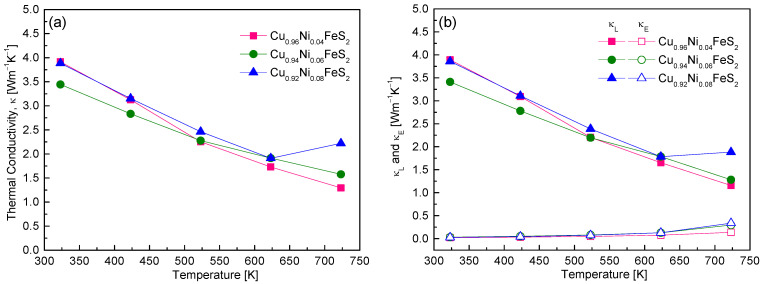
Temperature dependence of thermal conductivities of Cu_1−x_Ni_x_FeS_2_: (**a**) total thermal conductivity and (**b**) lattice and electronic thermal conductivity.

Figure 9 presents the thermal conductivity behavior of Cu_1−x_Ni_x_FeS_2_, highlighting the impact of Ni doping on phonon and electronic transport properties. Across all compositions, the total thermal conductivity decreased with the increase in temperature, a typical characteristic of crystalline semiconductors where Umklapp phonon–phonon scattering becomes dominant at elevated temperatures [18]. Among the samples, Cu_0.96_Ni_0.04_FeS_2_ exhibited the lowest thermal conductivity of 1.29 Wm^−1^K^−1^ at 723 K, which is slightly lower than the previously reported minimum value of 1.32 Wm^−1^K^−1^ for undoped CuFeS_2_ [7,16]. To better understand the origins of this reduction, the total thermal conductivity was separated into electronic (κ_E_) and lattice (κ_L_) contributions (Figure 9b). The electronic thermal conductivity was estimated using the Wiedemann–Franz law (κ_E_ = LσT), where the Lorenz number L was calculated via the single parabolic band model [18]. Subtracting κ_E_ from the total thermal conductivity yielded κ_L_. The results showed that κ_E_ was significantly lower than κ_L_ for all doping levels, confirming that thermal transport in Cu_1−x_Ni_x_FeS_2_ is predominantly governed by phonons. This aligns with the findings of Lefevre et al. [22], who reported that, in doped chalcopyrites, the lattice contribution is the major component of the thermal conductivity, and the influence of doping on κ_E_ is generally minor. Interestingly, κ_E_ did increase with higher Ni doping, consistent with the observed rise in carrier concentration and electrical conductivity. For instance, the κ_E_ value for Cu_0.92_Ni_0.08_FeS_2_ reached 0.34 Wm^−1^K^−1^ at 723 K. Nonetheless, even at this level, it remained well below κ_L_, reaffirming the phonon-dominated nature of thermal transport. The temperature and composition trends of κ_L_ followed those of total κ, indicating that Ni atoms substituted at Cu sites acted as efficient phonon-scattering centers. This phonon scattering is likely due to mass and strain field fluctuations introduced by the Ni dopants, which disrupt the regular lattice vibrations and impede heat transport. Cu_0.96_Ni_0.04_FeS_2_ achieved the lowest κₗ value of 1.16 Wm^−1^K^−1^ at 723 K. However, in Cu_0.92_Ni_0.08_FeS_2_, κ_L_ slightly increased above 623 K, which is attributed to bipolar conduction—a phenomenon where both electrons and holes contribute to thermal transport at high temperatures—counteracting the typical phonon scattering-induced decline. When compared to other doped chalcopyrites, Ni-doped CuFeS_2_ exhibits moderately low thermal conductivity. Ge et al. [16] achieved a significantly lower κ_L_ of 0.79 Wm^−1^K^−1^ at 723 K for Cu_0.92_In_0.08_FeS_2_, approaching the theoretical minimum of 0.74 Wm^−1^K^−1^. This reduction is attributed to the strong phonon-scattering effect of In atoms. Similarly, Ge et al. [18] showed that Ag doping reduced κ_L_ to 1.2 Wm^−1^K^−1^ in Cu_0.88_Ag_0.12_FeS_2_ due to the mass contrast introduced by Ag atoms. Ge et al. [19] reported that secondary phases such as FeS_2_ and CdS formed in Cu_0.92_Cd_0.08_FeS_2_ also served as phonon-scattering centers, reducing κ to ~1 Wm^−1^K^−1^. In contrast, Xie et al. [20] found that, in Cu_1−x_Zn_x_FeS_2_, Zn atoms and ZnS nanoparticles provided less effective scattering, resulting in a higher minimum κ of ~2 Wm^−1^K^−1^ for Cu_0.92_Zn_0.08_FeS_2_. In the present study, Ni doping in chalcopyrite effectively reduces thermal conductivity primarily through enhanced phonon scattering, though its impact is somewhat less pronounced than that achieved by In, Ag, or Cd doping. Nonetheless, it presents a balanced approach by simultaneously maintaining acceptable electronic transport properties, making it a viable strategy for thermoelectric performance optimization in chalcopyrite systems.

**Figure 10:**
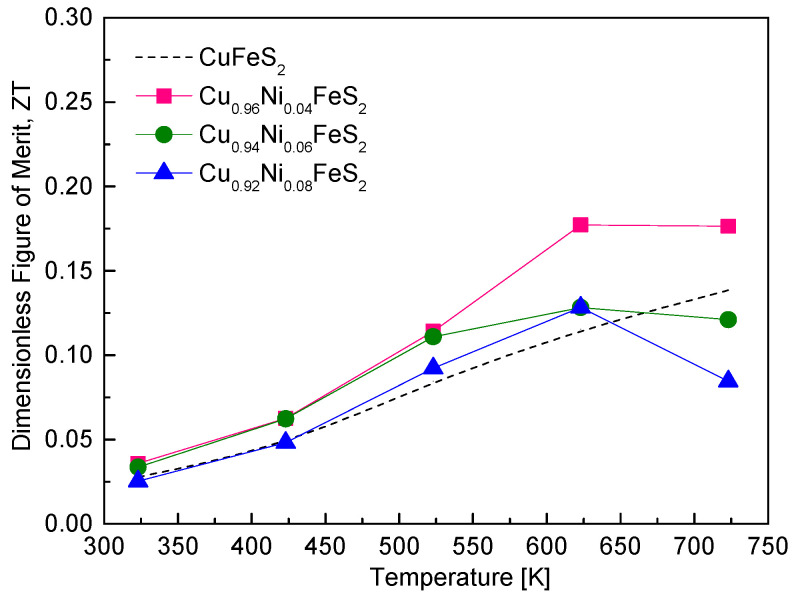
Dimensionless figure of merit for Cu_1−x_Ni_x_FeS_2_. For comparison, the ZT values of undoped chalcopyrite were presented [18].

Figure 10 illustrates the thermoelectric figure of merit (ZT) of Cu_1−x_Ni_x_FeS_2_ as a function of temperature. The ZT values for all samples increased with the rising temperature due to the enhanced power factor and reduced lattice thermal conductivity at elevated temperatures. Among the compositions, Cu_0.96_Ni_0.04_FeS_2_ exhibited the highest ZT value of 0.18 in the temperature range of 623–723 K, representing a moderate improvement over undoped CuFeS_2_, which has a ZT of 0.14 at 723 K [18]. This enhancement reflects the beneficial effect of Ni doping, where increased electrical conductivity, due to higher carrier concentration, compensates for the slight reduction in the Seebeck coefficient and contributes to improved PF and ultimately higher ZT. However, increasing the Ni doping level beyond 4 at.% (i.e., x > 0.04) led to a decrease in ZT, primarily due to a drop in the power factor. Although higher doping levels enhanced electrical conductivity further, the accompanying reduction in the Seebeck coefficient and the onset of bipolar conduction at high temperatures negatively impacted the thermoelectric efficiency. When compared to other doped CuFeS_2_-based chalcopyrites, the ZT values achieved in this study are modest. For instance, Ge et al. [16,18] reported significantly higher ZT values of 0.36 at 723 K for Cu_0.92_In_0.08_FeS_2_ and 0.32 at 600 K for Cu_0.88_Ag_0.12_FeS_2_. These compositions were synthesized using the melting–grinding–spark plasma sintering (SPS) process, which is known to produce dense, fine-grained microstructures that are effective at scattering phonons, thus lowering thermal conductivity and enhancing ZT. Xie et al. [20] achieved a ZT of 0.26 at 630 K for Cu_0.92_Zn_0.08_FeS_2_, synthesized using melting followed by plasma-activated sintering (PAS). Notably, even though the Zn solubility limit was x = 0.03, excess Zn led to the formation of ZnS nanoparticles, which acted as efficient phonon-scattering centers and reduced lattice thermal conductivity, boosting ZT. Similarly, Berthebaud et al. [21] observed a ZT of 0.22 at 675 K for Cu_0.94_Co_0.06_FeS_2_ prepared by a combination of cold pressing, solid-state reaction, and SPS, further emphasizing the role of process-induced microstructural control in improving thermoelectric performance. Ge et al. [19] demonstrated the highest reported ZT among these studies, achieving a value of 0.39 at 723 K for Cu_0.92_Cd_0.08_FeS_2_. This remarkable enhancement was attributed to the introduction of multi-scale defects via a complex synthesis route involving melting, quenching, annealing, high-energy ball milling, and PAS. These multi-scale defects were highly effective in scattering a broad spectrum of phonons, significantly lowering lattice thermal conductivity while preserving good electrical transport. Additionally, Lefevre et al. [22] reported a ZT of 0.20 at 623 K for Cu_0.97_Mn_0.03_FeS_2_, synthesized using melting–annealing–SPS. They proposed that d-shell spin disorder scattering, introduced by Mn atoms, played a crucial role in improving ZT by disrupting phonon transport without severely affecting carrier mobility.

## 4. Conclusions

N-type chalcopyrite compounds, Cu_1−x_Ni_x_FeS_2_ (0.04 ≤ x ≤ 0.08), were successfully synthesized through mechanical alloying followed by hot pressing, and their structural and thermoelectric properties were systematically investigated. X-ray diffraction analysis confirmed the formation of single-phase tetragonal chalcopyrite structures for all compositions. Ni substitution at Cu sites led to a slight contraction in lattice constants, consistent with the smaller atomic radius of Ni compared to Cu, and indicative of successful incorporation into the crystal lattice. From a transport perspective, Ni doping effectively increased the electron concentration, leading to enhanced electrical conductivity across the measured temperature range. The negative Seebeck coefficients confirmed n-type conduction behavior, which aligns with the electron-donating role of Ni in this system. Among the compositions, Cu_0.96_Ni_0.04_FeS_2_ demonstrated the most favorable balance between electrical conductivity and Seebeck coefficient, resulting in a peak power factor of 0.50 mWm^−1^K^−2^ at 623 K. Simultaneously, this composition exhibited the lowest total thermal conductivity of 1.29 Wm^−1^K^−1^ at 723 K, primarily due to suppressed lattice thermal conductivity. The reduction in lattice thermal conductivity can be attributed to enhanced phonon scattering induced by mass and strain field fluctuations from the Ni substitution. Compared to undoped CuFeS_2_, the thermoelectric performance of Cu_0.96_Ni_0.04_FeS_2_ was significantly improved, achieving a maximum ZT of 0.18 in the temperature range of 623–723 K. This moderate enhancement in ZT demonstrates that Ni is an effective n-type dopant for CuFeS_2_. The relatively straightforward synthesis route and the absence of secondary phases in the Ni-doped chalcopyrite compounds highlight the practical advantages of this doping approach.

## Figures and Tables

**Table 1:** Relative densities, lattice parameters, and crystallite sizes of Cu_1−x_Ni_x_FeS_2_ prepared via MA–HP process.

Specimen	Relative Density [%]	Lattice Parameter			Crystallite Size [nm]
		a [nm]	c [nm]	c/a	
Cu_0.96_Ni_0.04_FeS_2_	98.3	0.52913 (2)	1.04351 (8)	1.9722	85
Cu_0.94_Ni_0.06_FeS_2_	99.3	0.52918 (5)	1.04255 (2)	1.9705	47
Cu_0.92_Ni_0.08_FeS_2_	99.2	0.52879 (6)	1.04184 (2)	1.9705	36

## Data Availability

The original contributions presented in this study are included in the article; further inquiries can be directed to the corresponding author.
